# Field-Evolved Resistance to Bt Cry Toxins in Lepidopteran Pests: Insights into Multilayered Regulatory Mechanisms and Next-Generation Management Strategies

**DOI:** 10.3390/toxins18020060

**Published:** 2026-01-25

**Authors:** Junfei Xie, Wenfeng He, Min Qiu, Jiaxin Lin, Haoran Shu, Jintao Wang, Leilei Liu

**Affiliations:** 1 Center of Applied Biotechnology, School of Life Sciences and Technology, Wuhan University of Bioengineering, Wuhan 430415, China; jfxie@whsw.edu.cn (J.X.); hwenfeng2023@163.com (W.H.); qm2000@webmail.hzau.edu.cn (M.Q.); linjiaxin@webmail.hzau.edu.cn (J.L.); 102510805@hbut.edu.cn (H.S.); wjt217@126.com (J.W.); 2 College of Plant Science and Technology, Huazhong Agricultural University, Wuhan 430070, China; 3 School of Life and Health Sciences, Hubei University of Technology, Wuhan 430068, China

**Keywords:** *Bacillus thuringiensis*, cry toxin resistance, receptor gene regulation, ceRNA networks, sustainable pest management

## Abstract

Bt Cry toxins remain the cornerstone of transgenic crop protection against Lepidopteran pests, yet field-evolved resistance, particularly in invasive species such as *Spodoptera frugiperda* and *Helicoverpa armigera*, can threaten their long-term efficacy. This review presents a comprehensive and unified mechanistic framework that synthesizes current understanding of Bt Cry toxin modes of action and the complex, multilayered regulatory mechanisms of field-evolved resistance. Beyond the classical pore-formation model, emerging evidence highlights signal transduction cascades, immune evasion via suppression of Toll/IMD pathways, and tripartite toxin–host–microbiota interactions that can dynamically modulate protoxin activation and receptor accessibility. Resistance arises from target-site alterations (e.g., *ABCC2/ABCC3*, *Cadherin* mutations), altered midgut protease profiles, enhanced immune regeneration, and microbiota-mediated detoxification, orchestrated by transcription factor networks (GATA, FoxA, FTZ-F1), constitutive MAPK hyperactivation (especially MAP4K4-driven cascades), along with preliminary emerging findings on non-coding RNA involvement. Countermeasures now integrate synergistic Cry/Vip pyramiding, CRISPR/Cas9-validated receptor knockouts revealing functional redundancy, Domain III chimerization (e.g., Cry1A.105), phage-assisted continuous evolution (PACE), and the emerging application of AlphaFold3 for structure-guided rational redesign of resistance-breaking variants. Future sustainability hinges on system-level integration of single-cell transcriptomics, midgut-specific CRISPR screens, microbiome engineering, and AI-accelerated protein design to preempt resistance trajectories and secure Bt biotechnology within integrated resistance and pest management frameworks.

## 1. Introduction

*Bacillus thuringiensis* (Bt), a Gram-positive bacterium, produces insecticidal crystal proteins (Cry and Cyt) and vegetative insecticidal proteins (Vips) that are highly toxic to Lepidopteran, Coleopteran, and Dipteran pests while remaining safe and specific to target insects [1]. Cry proteins, the most widely deployed, have been incorporated into genetically modified (GM) crops since 1996, representing the most successful application of Bt technology [2]. These Bt crops have effectively controlled key Lepidopteran pests, including fall armyworm (*Spodoptera frugiperda*), cotton bollworm (*Helicoverpa armigera*), cutworm (*Spodoptera litura*), and diamondback moth (*Plutella xylostella*), while substantially reducing chemical insecticide use [1,3]. However, widespread adoption has driven rapid resistance evolution in Lepidopteran pests, with field-resistant populations now reported in multiple countries, posing a serious threat to the sustainability of Bt crops [2,4].

The invasive fall armyworm exemplifies this challenge: since 2016, it has spread to over 60 countries [5], entering China via Yunnan in 2019 and rapidly reaching major grain-producing regions [6]. Its voracious larvae can cause severe yield losses. Resistance often involves altered expression of Bt toxin receptor genes, commonly through mutations in regulatory elements that downregulate receptor levels [7]. Elucidating these mechanisms and their regulatory pathways is therefore essential for delaying resistance evolution.

Decades of molecular entomology research, coupled with breakthroughs in genome editing and transcriptomics, have ushered Bt toxin receptor studies into a new era of precise regulatory dissection [8]. The widespread adoption of these advanced tools has not only illuminated the genetic basis of Bt resistance but also opened new avenues for innovative resistance management strategies [1]. Notably, CRISPR/Cas9 enables nucleotide-level mutagenesis of receptor genes, allowing rigorous functional validation and confirmation of their critical roles in resistance evolution [9]. Simultaneously, high-throughput transcriptomic profiling under Bt toxin exposure, combined with systematic bioinformatics, comprehensively identifies differentially expressed genes and elucidates associated regulatory pathways and networks [2,10].

Despite progress, gaps persist in fully mapping these networks. Bt bioinsecticides remain a cornerstone of environmentally sound pest control. This review focuses on three key areas: (1) molecular regulation of receptor gene expression in Lepidopteran species; (2) genetic and epigenetic factors driving Bt resistance; and (3) resistance management strategies derived from mechanistic insights. By integrating these aspects, we aim to provide a robust mechanistic framework for integrated pest management and contribute to the long-term efficacy of Bt biotechnology within broader integrated resistance management (IRM) strategies, which most effectively also incorporate refugia and other complementary measures. This review not only synthesizes molecular mechanisms of Bt resistance but also emphasizes their translational relevance to resistance monitoring and management strategies.

## 2. Bt Toxin Mechanism of Action

Bt Cry toxins (also known as δ-endotoxins) are insecticidal pore-forming proteins featuring a conserved three-domain structure [11]. Domain I, a bundle of seven α-helices, serves as the pore-forming domain; its hydrophobic hairpin (α-helices 4 and 5) inserts into the midgut epithelial membrane to generate cation-permeable pores. Domain II, formed by three antiparallel β-sheets in a prism-like structure, contains exposed variable loops essential for specific binding to midgut receptors and insect host specificity. Domain III, with a β-sandwich (“jelly-roll”) topology, contributes to receptor recognition, binding stability, and membrane insertion [12].

**Figure 1:**
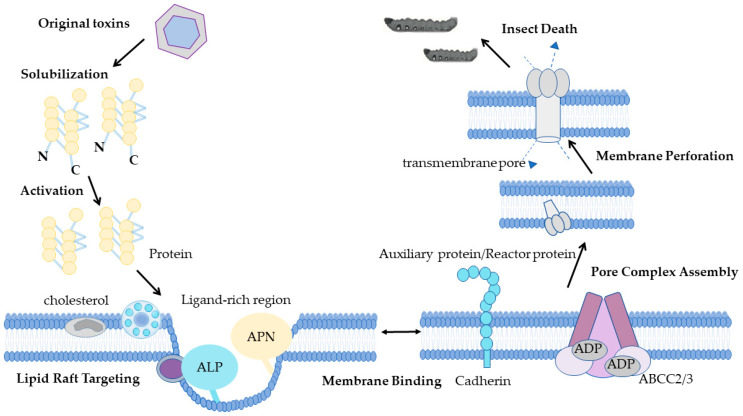
Model of midgut perforation formation.

The insecticidal action of Bt Cry proteins has traditionally been explained by two major models. The midgut pore formation model (Figure 1) holds that Cry toxins, after protoxin activation, bind specific receptors (primarily cadherins) on insect midgut epithelium, oligomerize, and insert into the membrane to form cation-permeable pores, causing osmotic lysis and insect death (Figure 1) [13,14,15]. Cry1A protoxin itself binds cadherin with high affinity, and midgut proteases generate two distinct oligomeric forms: protoxin-derived oligomers are thermally stable and form high-open-probability channels, whereas activated-toxin oligomers are less stable and less efficient at membrane insertion; both, however, insert into brush-border vesicles [16,17]. Interspecies variation in susceptibility may reflect differences in protoxin-to-toxin conversion rates governed by midgut protease profiles [18].

An alternative signal transduction model proposes that toxin oligomerization activates G-protein-coupled receptor cascades and cAMP-dependent pathways, leading to ion channel dysregulation and cytoskeletal collapse independent of pore formation [19]. These two models share identical initial steps (protoxin solubilization, proteolytic activation, and cadherin binding), but diverge in downstream events; current consensus supports pore formation as the primary mechanism of midgut cell disruption, while signal transduction may contribute complementarily under specific conditions or in certain cell types [15].

Merging evidence has revealed a third paradigm—the immune evasion model—in which Cry toxins suppress Toll and IMD pathways; in *Spodoptera exigua*, Cry1Ac exposure downregulates antimicrobial peptide synthesis via epigenetic modifications, compromising innate immunity [20]. This model likely operates in parallel with pore formation and signal transduction, as immune suppression may facilitate secondary bacterial septicemia or enhance overall toxicity in a microbiome-dependent context, rather than serving as an exclusive lethal pathway [21].

Recent studies have shifted focus to tripartite interactions among Cry toxins, host physiology, and gut microbiota [21]. Gut bacteria can profoundly modulate toxicity: some degrade protoxins via extracellular proteases, while others enhance solubilization, activation, or exert immunomodulatory effects [22], certain taxa even compete for cadherin-like or alkaline phosphatase receptors, disrupting toxin oligomerization and pore formation [23]. These findings demonstrate that Cry protein toxicity arises from intricate tri-directional networks integrating toxin structure-function, host-pathogen coevolution, and microbiome-mediated reprogramming. Resolving this context-dependent complexity requires holistic approaches combining entomopathology, microbial community profiling, and multi-omics analyses to guide microbiome-aware resistance management for overcoming resistance and ensuring sustainable Bt-based pest management.

## 3. Mechanisms of Bt Toxin Resistance in Lepidopteran Insects

### 3.1. Target-Site Resistance

Bt toxin insecticidal activity depends on binding to specific midgut receptors. Resistance arises from reduced binding affinity, genetic mutations, or lowered expression [24]. Examples include a single amino acid mutation in *H. armigera CAD* disrupting membrane localization [25]; cadherin variants in *Pectinophora gossypiella* conferring field-evolved Cry1Ac resistance [26]; down-regulation of *CAD1*/*CAD2* in *Chilo suppressalis* increasing Cry1Ac/Cry2A resistance [27]. CRISPR/Cas9 knockout of *HaABCA2* enhancing Cry2Aa/Cry2Ab resistance [28], RNAi suppression of *ABCG1* in *P. xylostella* elevating Cry1Ac resistance [9], and an amino acid insertion in *BmABCC2* linked to Cry1Ab/Cry1Ac resistance [29]. Reduced expression of ABC transporters or cadherin variants, a hallmark of target-site resistance, blocks the core pore formation mechanism by preventing toxin oligomerization and membrane insertion.

Recent proteomic studies have identified numerous auxiliary proteins beyond classical receptors (CAD, ABC transporters, ALP, APN). These include additional membrane-associated proteins such as GPI-anchored enzymes (e.g., APNs and ALPs), certain lipid raft-associated components, and other potential auxiliary factors that may participate in multimeric complex formation, membrane microdomain reorganization, and toxin trafficking [30,31]. Post-translational modifications (phosphorylation, ubiquitination) dynamically regulate receptor-complex assembly. This expanding interactome challenges the traditional linear toxin–receptor model and shifts the paradigm toward three-dimensional networks. Future studies should integrate cryo-electron tomography, RNAi screens, and comparative proteogenomics across resistant/susceptible strains [10,30]. to clarify how coordinated changes in auxiliary proteins drive field-evolved resistance and to guide next-generation receptor-engineering strategies.

### 3.2. Metabolic Resistance-Midgut Protease Activity

Changes in the activity of midgut proteases in Lepidopteran insects are an important mechanism for resistance development. The serine protease activity in the European corn borer (*Ostrinia nubilalis*) resistant strain is significantly lower than that in the susceptible strain [32]. The altered composition of midgut proteases in the resistant population of the tobacco budworm (*Heliothis virescens*) is directly related to the decreased activation of protoxins [33], yet the relative contribution of individual proteases within this complex enzymatic network remains unclear. The absence of trypsin in the resistant strain of the Indian meal moth (*Plodia interpunctella*) prevents the activation of protoxins [34]. A mutation in the promoter of a trypsin gene in the cotton bollworm enhances resistance to Cry1Ac [35], but whether this regulatory change alone is sufficient to explain high-level resistance across developmental stages and environmental conditions requires further investigation. However, the high resistance of the Cry1Ac-resistant strain (NIL-R) of the diamondback moth does not appear to be directly related to changes in midgut protease activity [36]. Given the presence of numerous trypsin and chymotrypsin genes in its genome [37], this mechanism requires further validation. It is worth noting that, according to traditional theory, Cry protoxins must be converted into activated toxins to exert toxicity [14]. However, some resistant insects are still sensitive to Cry1Ab/c protoxins, suggesting that Bt proteins may exert secondary toxic effects through protoxins or their fragments [38,39]. Recent studies have shown that chitin synthase *CHS2* in the midgut can mediate high-level resistance to Vip3Aa toxin in at least three species of Lepidopteran pests [40], indicating that protease activity plays a key role in the evolution of resistance [41,42]. Altered midgut protease profiles underlying metabolic resistance interfere with protoxin activation, a prerequisite for the pore formation mechanism, thereby reducing toxin availability for membrane insertion. Beyond mutations and transcriptional regulation, midgut protease activity is finely tuned by endocrine (ecdysteroid/juvenile hormone-MAPK-epigenetic axes) [43,44,45], nutritional, and environmental cues. Abiotic stressors further reprogram activity through redox and ubiquitin-proteasome pathways.

This multilayered regulation positions midgut proteases as a central hub integrating genetic, physiological, and ecological factors to drive metabolic resistance [46]. Future progress requires integrated approaches-combining hormone receptor knockouts, artificial diet assays, activity-based profiling, multi-omics (phosphoproteomics, metabolomics), and ex vivo midgut simulations—to dissect context-specific interactions and enable predictive modeling of resistance trajectories in agroecosystems [44], but its relative importance likely varies among species, toxin types, and resistance backgrounds, underscoring the need for integrative and comparative studies.

### 3.3. Immune-Related Resistance

Insects can develop Bt resistance by enhancing detoxification and immune responses. Elevated carboxylesterase or acetylcholinesterase activity accelerates toxin degradation in several Lepidopteran species [21,34]. Midgut microbiota sustain a primed immune state that enhances survival under Bt challenge [47], while *HaDNV-1* infection in cotton bollworm (*H. armigera*) significantly increases tolerance to low-dose Cry toxins by promoting growth and reducing toxin efficacy [48,49]. Conversely, silencing key immune genes markedly heightens susceptibility [50], confirming that immune activation is a pivotal resistance mechanism.

Emerging studies reveal that the insect immune system constitutes a multilayered defense against Bt toxins. First, Toll and IMD pathways induce antimicrobial peptides, which contribute to gut immune homeostasis and may indirectly modulate host responses to pore-forming toxins [51,52]. Second, immune signaling strengthens the peritrophic matrix and upregulates ABC transporters, forming physical and active barriers to toxin penetration [53]. Third, sustained immune activation drives rapid regeneration of damaged epithelium through stem cell proliferation [54].

This integrated immune defense—encompassing humoral responses, structural reinforcement, and regenerative repair—operates synergistically with detoxification and microbiota-mediated mechanisms [46]. Future research should employ midgut-specific RNAi/CRISPR screening, single-cell transcriptomics, and live imaging of immune dynamics to dissect pathway contributions and crosstalk [55]. Such insights will inform the potential development of immune-suppressing synergists, thereby restoring Bt efficacy and extending the durability of Bt-based pest control [56].

## 4. Regulatory Mechanisms of Receptor Gene Expression and Resistance

### 4.1. Transcriptional Regulation by Transcription Factors

Transcriptional regulation of Bt Cry toxin receptor genes in Lepidopteran insects is orchestrated primarily by specific transcription factors (TFs) binding to promoter regions [57]. Among these, Forkhead box (Fox) proteins play pivotal roles. FoxA, a pioneer factor, directly opens compacted chromatin by displacing nucleosomes, thereby facilitating access for other regulators [58]. In Lepidopteran species, FoxA upregulates *ABCC2* and *ABCC3* expression in Sf9 cells, and reduced FoxA levels correlate with Bt resistance. In the resistant cotton bollworm strain LF60, mutations in FoxA-binding sites within the *HaABCC3* promoter drive receptor downregulation [59].

FoxO regulates diverse physiological processes and intersects with the MAPK signaling cascade in Lepidoptera [60,61,62]. The hormones 20-hydroxyecdysone (20E) and juvenile hormone (JH) activate MAPK pathways (including p38, JNK, and ERK), which in turn modulate multiple Cry receptor genes (*ALP*, *APN1*, *APN3a*, *ABCB1*, *ABCC2*, *ABCC3*, *ABCG1*) [53,62,63,64]. In diamondback moth, constitutive MAPK activation—often triggered by retrotransposon insertion in the MAPK4 promoter—downregulates functional Cry1Ac receptors while upregulating non-receptor paralogs (e.g., *APN5/6*, *PxABCC1*), potentially reducing fitness costs of resistance [53].

The GATA zinc-finger family, particularly GATAe, is highly enriched in insect midguts and governs tissue differentiation, stem-cell homeostasis, and immune defense [65,66,67]. Recent work identified GATAe, GATAd, and FTZ-F1 as direct regulators of Cry1Ac receptors in diamondback moth and cotton bollworm [68,69]. In fall armyworm, SfGATAe specifically activates *SfABCC2* in midgut cells; its silencing markedly reduces receptor expression and Cry1Ac susceptibility [6]. These findings highlight synergistic TF networks (Table 1, e.g., GATAe with CDX, Sox21, or Notch signaling) that fine-tune receptor levels [70].

Emerging evidence expands this regulatory landscape to include metamorphosis- and stress-responsive TFs (bZIP, C2H2 zinc fingers, Hox-POU, and HSF families), whose activity is modulated by hormonal (20E), nutritional (TOR), and environmental cues. This multilayered control establishes a dynamic equilibrium integrating developmental, metabolic, and epigenetic signals. Future studies should leverage midgut-specific inducible CRISPR knockouts, ChIP-seq, Hi-C, single-cell transcriptomics, and phosphoproteomics to map TF-promoter-enhancer networks and their post-translational modifications. Such integrative approaches will pinpoint resistance-associated transcriptional hubs, enabling precision strategies, such as TF-targeted RNAi or small-molecule modulators—to restore receptor expression and prolong the efficacy of Bt crops. However, the application of RNAi-based traits faces practical challenges, including variable delivery efficiency, environmental instability, and inconsistent field-level durability, which may constrain their standalone use for resistance management [2].

### 4.2. Signal Transduction Pathway Regulation

High-level resistance to Bt Cry toxins in Lepidopteran insects is tightly linked to altered signal transduction, with the MAPK pathway emerging as a central regulator. In *Choristoneura fumiferana* CF1 cells, Cry1A sensitivity correlates with MAPK rather than AC/PKA signaling [72]. Silencing MAPK-p38 or MAPK-INVK in rice stem borer larvae dramatically increases susceptibility [73]. In diamondback moth, MAPK-mediated phosphorylation of FTZ-F1 downregulates multiple receptor genes, conferring near-complete Cry1Ac resistance [74]. Zhang Youjun’s team demonstrated that a retrotransposon-driven constitutive activation of PxMAP4K4 hyperactivates MAPK signaling, represses PxGATAd, and consequently suppresses key receptors (ALP, ABCC2/3), while silencing PxGATAd restores susceptibility [53,62,63,64]. These findings delineate a well-defined regulatory cascade: constitutive MAPK hyperactivation represses GATAd, which in turn downregulates key midgut receptor genes, culminating in high-level Cry1Ac resistance. The MAPK mechanism appears more prevalent in laboratory-selected strains, potentially overemphasized due to selection pressure, while its field prevalence and contribution to practical resistance remain understudied and possibly overstated.

Beyond these well-characterized MAPK cascades, preliminary evidence suggests broader signaling integration [53]. The PI3K-Akt-FoxO axis and NF-κB modules, primarily known for apoptosis and immune regulation [36], may indirectly influence receptor gene expression through phosphorylation-dependent TF shuttling and chromatin remodeling [75]. However, direct causal links in Bt resistance remain to be fully established [76]. Similarly, potential MAPK–PI3K crosstalk and feedback loops mediated by dual-specificity phosphatases warrant further validation [62]. Resolving these complex networks demands targeted functional tools: midgut-specific CRISPR interference of core kinases, live-cell phosphoproteomics, and pathway-selective inhibitors. Such approaches will clarify the hierarchy and context-dependency of signaling events driving receptor downregulation, paving the way for potential rationally designed, pathway-specific synergists that restore Bt efficacy in resistant field populations.

### 4.3. Regulation by Competitive Endogenous RNAs (ceRNAs)

**Figure 2:**
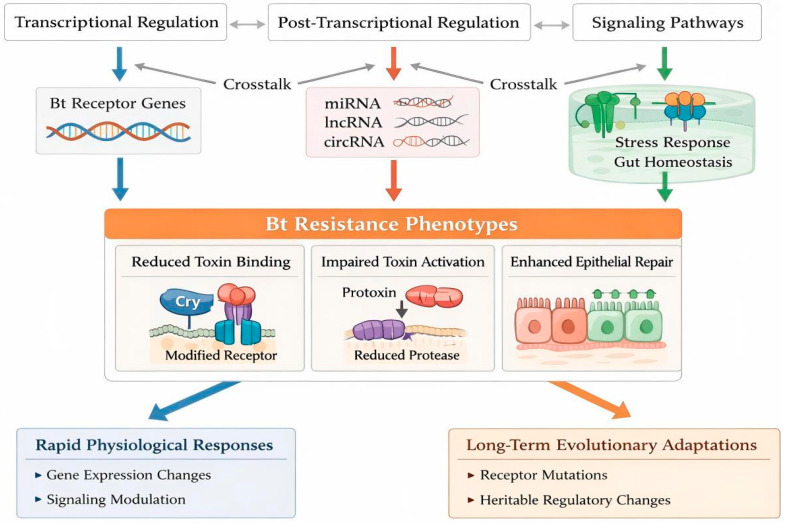
An integrated regulatory framework underlying Bt resistance in lepidopteran insects. This schematic illustrates how multiple regulatory layers collectively shape Bt resistance phenotypes. At the upstream level, transcriptional regulation, post-transcriptional regulation (mediated by miRNAs, lncRNAs, and circRNAs), and intracellular signaling pathways interact through extensive crosstalk to modulate the expression and function of key Bt receptor genes and gut physiological processes. These coordinated regulatory changes converge on central Bt resistance phenotypes, including reduced toxin–receptor binding, impaired protoxin activation due to altered midgut protease activity, and enhanced epithelial repair and gut homeostasis.

In recent years, non-coding RNAs (ncRNAs)-particularly microRNAs (miRNAs), long non-coding RNAs (lncRNAs), and circular RNAs (circRNAs)-have emerged as critical post-transcriptional and epigenetic regulators of Bt resistance in Lepidopteran pests [77]. miRNAs exert profound effects on insect development, immunity, and xenobiotic detoxification by binding to the 5′ UTR, CDS, or 3′ UTR of target mRNAs, triggering degradation or translational repression (Figure 2) [78,79].

Multiple microRNAs (miRNAs) have been shown to modulate Bt toxin susceptibility in Lepidopteran pests by targeting key detoxification and receptor-related genes, forming a conserved yet diverse regulatory network across species (Table 2). In diamondback moth, upregulated miR-310 and miR-8510a-3p target PxABCG20 and PxABCG3, respectively, enhancing tolerance in susceptible strains [7,77,78]. Similarly, miR-998-3p represses ABCC2 across cotton bollworm, beet armyworm, and diamondback moth, contributing to Cry1Ac resistance [80]. In resistant diamondback moth larvae, sublethal Cry1Ac exposure elevates PxTrypsin-9; miR-2b-3p directly suppresses this gene via its CDS, further increasing tolerance [81]. In rice stem borer, the miR-7322-5p/p38/Hsp19 axis modulates Cry1Ca sensitivity: miR-7322-5p downregulates p38, reducing Hsp19 phosphorylation and thereby decreasing susceptibility [60].

LncRNAs orchestrate gene expression at multiple levels and are broadly implicated in insect development, immunity, and dosage compensation [61]. Transcriptome profiling of Bt-exposed resistant and susceptible strains has identified 59 resistance-associated lncRNAs [81], with several highly expressed in resistant cotton bollworm [91]. Notably, the cadherin gene *PgCAD1* in pink bollworm produces both a receptor protein and an lncRNA; silencing the latter via siRNA significantly reduces Cry1Ac susceptibility, demonstrating positive regulation of cadherin expression by its own lncRNA [92,93].

**Figure 3:**
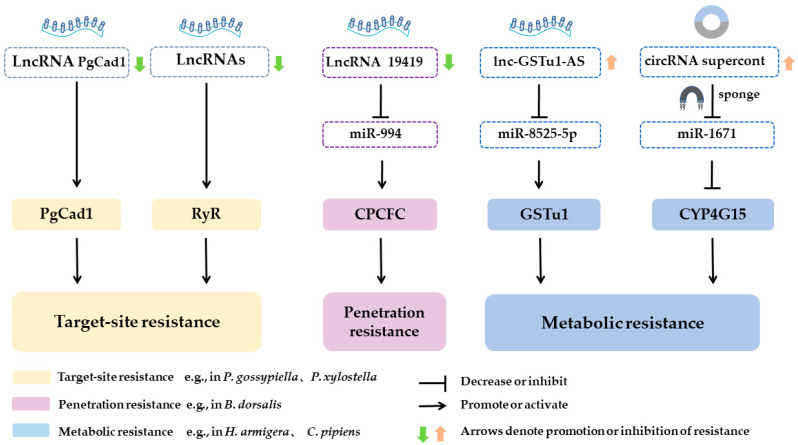
Mechanism of insecticide resistance mediated by lncRNA and circRNA in insects. The common mechanisms by which lncRNA/circRNA affect insecticide resistance include: (1) acting as a sponge for miRNAs, which affects the expression of target genes; (2) influencing the expression of target or downstream genes. Black arrows represent promotion or activation; black blunt arrows represent decrease or inhibition. The colored arrows denote regulatory relationships identified in specific insect species: gray arrows exemplify findings from the diamondback moth (*P. xylostella*) or the pink bollworm (*P. gossypiella*), primarily associated with target-site resistance; blue arrows exemplify findings from the oriental fruit fly (*B. dorsalis*), primarily associated with penetration resistance; green arrows exemplify findings from the cotton bollworm (*H. armigera*) or the mosquito (*C. pipiens*), primarily associated with metabolic resistance. The direction of the arrows indicates whether the function of the corresponding lncRNA/circRNA enhances or suppresses resistance in the specified pest.

Circular RNAs (circRNAs), derived from back-splicing of exons, introns, or intergenic regions [85,86], influence development, reproduction, and immunity [94]. Their covalently closed structure confers exceptional stability against exonucleases [95]. Both circRNAs and lncRNAs function as competing endogenous RNAs (ceRNAs), sponging miRNAs to relieve repression of detoxification enzymes and resistance-related targets—a mechanism central to metabolic and target-site resistance. LncRNAs also regulate cuticle protein genes, modulating cuticle thickness and insecticide penetration resistance (Figure 3) [96]. Moreover, the functional redundancy of receptors (e.g., ABCC2/3, APN1/3) often observed in resistant pests, which is fine-tuned by ceRNA networks, compensates for impaired toxin binding to maintain midgut integrity, thereby overriding the pore formation mechanism; simultaneously, ceRNA-mediated modulation of signal transduction cascades further weakens Cry toxin efficacy. Collectively, dissecting these intricate ncRNA–mRNA networks offers critical insights into the epigenetic and post-transcriptional control of Bt receptor expression and insecticide resistance in Lepidopteran pests [97]. In the BmNPV infection model, virus-derived vcircRNA-390 encodes an 81-aa peptide (VSP81) via IRES-driven translation; VSP81 suppresses the host RNAi antiviral pathway to facilitate viral replication, underscoring the proviral regulatory role of circRNAs in immune evasion [98].

The competitive endogenous RNA (ceRNA) mechanism was initially identified in pseudogenes, which contain highly conserved miRNA response elements (MREs) that competitively bind miRNAs to protein-coding genes [99]. Theoretically, any RNA molecule harboring miRNA binding sites can function as a ceRNA. Experimental evidence demonstrates that overexpression of long non-coding RNAs (lncRNAs) is essential for their “molecular sponge” function and the initiation of ceRNA regulatory pathways. For instance, overexpressed lncRNA-NEAT1 acts as a “sponge” for miR-377-3p, thereby promoting *E2F3* expression [99]. Circular RNAs (circRNAs) are particularly suitable as ceRNAs due to their unique characteristics, including covalently closed circular structures, predominant cytoplasmic localization, and non-coding nature [100]. Although the ceRNA mechanism has been extensively studied in human diseases, particularly in cancer research where lncRNA-mediated therapeutic resistance often involves direct interactions with transcription factors or RNA-binding proteins to regulate downstream gene expression [101], the specific mechanisms underlying transcription factor-lncRNA interactions and lncRNA-protein regulatory networks in insects remain to be further elucidated [102,103].

## 5. Strategies for Improving Resistance Management to Bt Toxins in Lepidopteran Insects

### 5.1. Synergistic Effects of Cry Toxins

Combinations of Cry toxins often exhibit strong synergism, substantially enhancing toxicity against target Lepidoptera and offering a practical strategy to delay resistance evolution [104]. Heterodimer formation between Cry1Aa and Cry1Ab/Cry1Ac markedly increases binding affinity to the ABCC2 transporter in *S. exigua* [104], while Cry1Ac + Cry1Fa displays pronounced synergism against *H. armigera*, far exceeding the efficacy of individual toxins [105,106]. Cry toxins also synergize with vegetative insecticidal proteins (Vip3Aa), which bind distinct midgut receptors and therefore have little or no cross-resistance with Cry proteins [107,108]. Notably, Vip3Aa enhances Cry9Aa toxicity against *Chilo suppressalis* by direct protein–protein interaction involving the exposed loop of domain II in Cry9Aa, with evidence suggesting Vip3Aa may serve as an alternate receptor [109]. These non-overlapping binding sites provide a robust approach for deploying Cry + Vip pyramided traits to prolong Bt efficacy in the field.

### 5.2. Gene Editing Technology

CRISPR/Cas9-mediated knockouts have greatly clarified the functional roles of midgut receptors and the genetic basis of Bt resistance in Lepidoptera. Disruption of cadherin in *H. armigera* confers high-level resistance to Cry1Ac [110,111,112], whereas *ABCC2* knockouts in *P. xylostella*, *H. armigera*, and *S. frugiperda* typically yield only moderate resistance compared with field-evolved mutations [112,113,114,115,116,117]. However, simultaneous knockout of *ABCC2* and *ABCC3* produces extremely high resistance to Cry1Ac/Cry1Ab in both *P. xylostella* and *H. armigera*, revealing substantial functional redundancy between these transporters [118].

In the invasive *S. frugiperda*, long-term Cry1Ab selection on an *SfABCC2*-null background still generated >300-fold resistance to Cry1F through novel *SfABCC2* mutant alleles, yet no cross-resistance to Vip3Aa was observed, highlighting receptor-specific resistance mechanisms and the sustained efficacy of Vip3Aa in newly invaded regions [119]. Among GPI-anchored receptors, *APN1* knockout in *H. armigera* does not alter susceptibility to activated Cry1Ac toxin [120,121], whereas *APN1* or *APN3* disruption in *P. xylostella* markedly reduces sensitivity to Cry1Ac protoxin, confirming their functional roles in protoxin intoxication [118]. Double *APN1*/*APN3* knockout elicits far greater resistance than single knockouts due to pronounced isoform redundancy, with mutants remaining more susceptible to Cry1Ab/Cry1Aa protoxins than to Cry1Ac—a pattern consistent with findings in *Manduca sexta* showing stronger *ALP*/*APN* contributions to Cry1Ac protoxin toxicity [118,122]. Accordingly, quadruple *APN1/APN3/ABCC2/ABCC3* knockout in *P. xylostella* confers dramatically enhanced resistance to Cry1Ac protoxin, indicating cooperative action among these receptors in the intoxication pathway [118].

**Figure 4:**
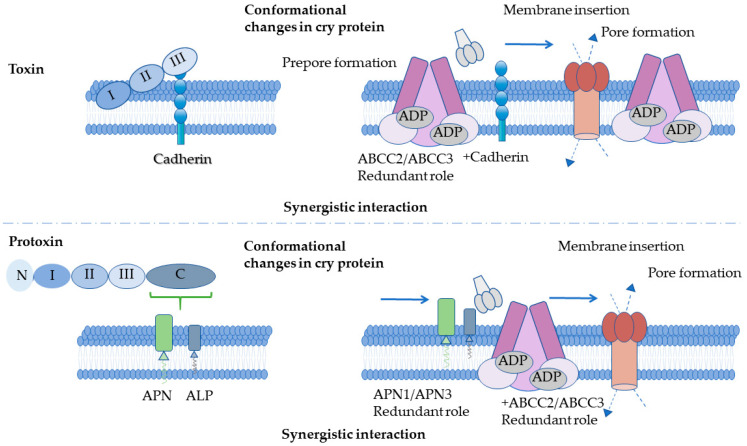
Dual Action Modes of Cry-Activated Toxins and Protoxins. The upper panel depicts activated toxin binding to Cadherin, inducing conformational changes in Cry protein, prepore formation with redundant roles of ABCC2/ABCC3 and Cadherin, and synergistic membrane insertion leading to pore formation. The lower panel illustrates protoxin binding to APN and ALP, conformational changes, redundant roles of APN1/APN3 and ABCC2/ABCC3, and synergistic interaction during membrane insertion and pore formation.

Gene editing has also elucidated the “dual mode of action” of Cry toxins: cadherin knockout in *H. armigera* primarily impairs activated Cry1Ac toxicity while sparing protoxin sensitivity, whereas ABCC2/ABCC3 are essential for both forms [109]. In cells co-expressing cadherin with ABCC2/3, activated toxins (but not protoxins) display synergistic receptor binding (Figure 4) [112]. Nevertheless, potential off-target effects and genetic compensation in CRISPR/Cas9-edited lines may complicate phenotypic interpretation, particularly in polyploid or highly heterozygous Lepidopteran genomes. Consequently, *ABCC2/ABCC3* double knockout dramatically elevates resistance, underscoring the central role of these transporters in Cry1Ac toxicity across Lepidoptera [112,114,115,118]. However, complete knockout, while conferring high resistance, is also associated with severe fitness costs, suggesting that such alleles are unlikely to persist in natural populations [123].

### 5.3. Protein Engineering for Resistance-Breaking Bt Toxins

Domain III of Cry toxins plays a pivotal role in receptor recognition, binding stability, and insect specificity, making its exchange a powerful protein-engineering strategy to generate broad-spectrum or resistance-breaking variants [124]. Cry1Ac, Cry1Be, and Cry1Bg share high Domain III homology with Cry1Cb, facilitating successful chimerization [124]. By swapping Domain III among Cry proteins, researchers have produced mutants with dramatically enhanced toxicity, several of which have been commercialized in transgenic crops [124]. In Lepidoptera, the widely deployed Cry1A.105—a chimeric toxin comprising Domains I and II from Cry1Ab/Cry1Ac and Domain III from Cry1Fa—exhibits exceptionally high potency against *S. frugiperda*, primarily due to markedly improved binding affinity to ABCC2 transporters [125,126]. Similarly, a hybrid harboring Domains I/II of Cry1Ab and Domain III of Cry1Ca significantly increases toxicity toward *Agrotis* spp. [127]. In Coleoptera, the engineered Cry3.1Ab (Domains I/II from Cry3Aa and Domain III from Cry1Ab) displays substantially elevated activity against *Diabrotica virgifera virgifera* [124]. These examples demonstrate that Domain III exchange can expand receptor-binding spectra and overcome field-evolved resistance, offering a proven approach to prolong the efficacy of Bt crops against major Lepidopteran pests.

### 5.4. Improving Cry Toxicity Through In Vitro Evolution Technologies

Currently, only a limited number of Cry genes have been successfully introduced into transgenic crops for Lepidopteran pest control, including Cry1A.105, Cry1Ab, Cry1Ac, Cry1Fa, Cry2Ab, and Cry9C in crops such as corn, cotton, and soybean, as well as Cry3Bb, mCry3Aa, and eCry3.1Ab in corn [34]. To counter the evolution of resistance in target pests, it is critical to explore new bacterial sources or apply in vitro evolution technologies to improve Cry toxins and generate novel insecticidal proteins with high potency and unique modes of action [128]. Directed evolution and site-directed mutagenesis have successfully enhanced Cry toxin potency against target pests [129], supported by high-throughput screening platforms that rapidly evaluate large mutant libraries [124]. Among these, genome shuffling has produced Cry1Ca and Cry1Bj variants with markedly increased toxicity toward *S. frugiperda* and *H. armigera*, respectively, by recombining homologous sequences and selecting for improved receptor binding [130,131]. Similarly, shuffling of Cry1Bj with related Cry1 genes has enhanced toxicity against *H. armigera* [131]. Phage display has also proven effective for identifying Cry mutants with higher affinity to midgut receptors [129]. Advanced cell-free techniques, including ribosome display and phage-assisted continuous evolution (PACE), overcome these limitations by enabling rapid, continuous mutant generation and selection [132]. Notably, PACE-derived Cry1Ac variants targeting a novel cadherin-like receptor (TnCAD) in *Trichoplusia ni* exhibit up to 300-fold higher activity than the wild type, while retaining sufficient stability for practical use [133]. These engineered toxins not only restore susceptibility in resistant populations but also expand the insecticidal spectrum through altered receptor specificity [133].

Recent advances in machine learning-assisted protein design, particularly the integration of AlphaFold3 with directed evolution, are markedly accelerating Bt toxin optimization by accurately predicting toxin–receptor interactions and prioritizing high-affinity variants in large libraries [134,135]. For example, AlphaFold3-guided iterative evolution has enabled the rational design of stabilized Cry-like scaffolds with enhanced specificity against resistant Lepidopteran ABC transporters, reducing experimental screening cycles by up to 80% [136]. Collectively, integrating high-throughput evolution platforms with receptor-binding selection—and emerging AI tools—offers a robust and scalable strategy to develop next-generation Bt toxins with enhanced efficacy and durability against Lepidopteran pests [133]. Combined with complementary agronomic practices, including structured refugia, crop rotation, and integrated pest management, these approaches contribute to more robust resistance management frameworks in the field [2,137].

## 6. Translating Molecular Mechanisms to Management Practice

The molecular mechanisms of Bt resistance in Lepidopteran insects, including alterations in receptor binding (e.g., cadherin, ABCC2/ABCC3 mutations or knockouts), impaired protoxin activation, metabolic detoxification, and regulatory changes at transcriptional and post-transcriptional levels, provide critical insights for translating basic research into practical pest management strategies.

### 6.1. Detection and Monitoring

Current molecular diagnostic tools offer rapid and scalable alternatives to bioassays for resistance monitoring. For target-site resistance, including mutations in cadherin and ABCC2/ABCC3, qPCR and amplicon sequencing are widely used to quantify resistance allele frequencies and enable early detection in field populations, with proven application in pests such as *H. armigera* and *S. frugiperda* [2,138]. Compared with bioassays, which require many live insects and are time-consuming and less sensitive to early resistance, molecular approaches (qPCR/amplicon sequencing) are faster and more cost-effective for large-scale monitoring. While bioassays remain essential for confirming phenotypic resistance, molecular diagnostics provide complementary mechanistic insights [139]. Regarding post-transcriptional regulators, signatures of differential expression have been linked to metabolic and target-site resistance in insects (Figure 2). However, ceRNA networks currently serve primarily as research tools for understanding resistance evolution rather than routine early detection in field monitoring, due to the complexity of validation and lack of standardized, high-throughput assays for practical application [140,141].

### 6.2. Mechanism-Specific Refuge Optimization

Fitness costs associated with Bt resistance significantly influence the efficacy of refuge strategies, with higher costs delaying resistance evolution by disadvantaging resistant individuals on non-Bt hosts. Target-site resistance often incurs substantial fitness costs in Lepidoptera, supporting the use of structured refuges to promote dilution of resistance alleles [142]. Metabolic resistance (e.g., enhanced detoxification) often entails lower or variable fitness costs, necessitating larger refuges or complementary strategies such as toxin pyramiding. Accordingly, smaller refuges may be adequate for high-cost target-site resistance, whereas metabolic resistance requires broader refuges or integrated management approaches [143].

### 6.3. Resistance Management Prioritization and Decision Framework

Among resistance mechanisms, target-site alterations are often highly amenable to intervention via high-dose pyramided traits (e.g., Cry1A.105 + Cry1Fa), which exploit lack of cross-resistance and restore susceptibility. Metabolic resistance may be addressed through gene-edited or evolved toxins with novel binding specificity. Overall, toxin pyramiding with structured refuges offers the highest efficacy, lowest cost, and greatest feasibility, while fitness cost–based refuge adjustment provides moderate benefits. In contrast, ceRNA-targeted and advanced detection approaches remain less feasible and largely experimental [144]. These mechanism-informed approaches, building on established high-dose/refuge and pyramiding strategies, enhance the durability of Bt crops against Lepidopteran pests. Future integration of rapid molecular diagnostics and fitness cost assessments will further refine proactive resistance management [137,145].

## 7. Conclusions

The regulation of Bt toxin receptor gene expression in Lepidopteran insects represents a complex biological process with critical implications for pest resistance management and the sustainable application of Bt-based pest control strategies [146]. Our comprehensive review reveals that resistance evolution in Lepidopteran pests against Bt toxins is governed by a sophisticated interplay of molecular mechanisms, including ABCC2 mutations as a primary target-site alteration in *H. armigera*, metabolic adaptations such as enhanced esterase sequestration in *H. virescens*, and immune-related responses like hexamerin-mediated coagulation in *P. xylostella* [24,147,148]. These mechanisms are further modulated by intricate transcriptional networks, signal transduction pathways, and non-coding RNA interactions, highlighting the multifaceted nature of pest resistance.

We elucidate how transcription factors such as GATAe in *H. armigera* and GATAd in *P. xylostella* precisely orchestrate receptor gene expression [69], while the MAPK signaling pathway emerges as a central coordinator of resistance-related transcriptional reprogramming, as evidenced by MAP4K4 activation driving ABCC gene downregulation in diamondback moth [149]. The emergence of non-coding RNAs (ncRNAs) as key post-transcriptional regulators adds another layer of complexity, with ceRNA networks fine-tuning receptor gene expression through competitive interactions, exemplified by miR-998-3p-lncRNA modulation of ABCC2 in resistant strains [80]. These findings collectively underscore the importance of adopting a systems biology approach to fully understand the regulatory architecture governing Bt toxin receptor genes (Figure 2).

Our analysis also emphasizes the potential of cutting-edge technologies in addressing resistance challenges. Gene editing tools like CRISPR/Cas9 enable precise manipulation of receptor genes, offering insights into their functional roles, such as *HaCad* knockout confirming its role as a Cry1Ac receptor in cotton bollworm and opening avenues for developing resistance-breaking strategies [110]. The synergistic effects of Cry toxin combinations and the innovative application of in vitro evolution technologies further provide promising prospects for enhancing Bt toxin efficacy and durability in the field. Despite these advances, significant knowledge gaps persist regarding the complete regulatory networks of receptor genes and their dynamic interplay with pest physiological states and environmental factors. Key unresolved issues include the full mapping of ceRNA-miRNA interactions under field stress and the evolutionary fitness costs of MAPK-mediated adaptations in invasive species like *S. frugiperda*. Future research must integrate multi-omics approaches with ecological and evolutionary perspectives to predict resistance evolution trajectories and design more robust resistance management frameworks. By bridging these gaps, the scientific community can ensure the prolonged efficacy of Bt toxins, preserving their value as a cornerstone of green pest management in agricultural systems worldwide.

## Figures and Tables

**Table 1:** Summary of Lepidopteran Pest-Related Transcription Factors and Their Regulatory Roles in Bt Toxin Resistance.

Species	TFs	Regulated Object	Regulatory Mechanism	Signaling Pathway	Resistance Phenotype	References
*S. frugiperda*	SfGATAe	*SfABCC2*	Binds to key motifs in the *SfABCC2* promoter; knockdown reduces receptor expression	Notch, MAPK pathways	Reduced susceptibility to Cry1Ac toxin	[6]
*H. armigera*	HaGATAe	*HaABCC2*	Cooperates with CDX/Sox21 to regulate midgut differentiation and receptor expression	Midgut developmental pathway	Linked to resistance mechanisms	[70]
*H. armigera*	FoxA	*HaABCC2*, *HaABCC3*	Upregulates receptor expression via promoter binding; mutations cause downregulation	MAPK pathway	Enhanced resistance to Cry1Ac	[63]
*H. armigera*	CDX/Sox21	*ABCC2*/*ALP*	Cooperates with GATAe to regulate developmental genes and receptor stability	Wnt/β-catenin	Development-dependent resistance	[70]
*H. armigera*	EcR/USP	*HaALP*, *HaABCC3*	Ecdysone receptor complex controls developmental genes	20E signaling pathway	Dynamic receptor expression during larval-pupal transition	[63,70]
*P. xylostella*	FoxO	*PxALP*, *PxABCC2*	Phosphorylation-dependent suppression of receptor genes via MAPK interaction	MAPK4-JNK	Perfect Cry1Ac resistance	[62]
*P. xylostella*	FTZ-F1	*PxABCB1*, *PxABCC2*	Retrotransposon insertion activates constitutive expression, repressing receptor promoters	Retrotransposon	Low fitness cost resistance	[2]
*C. suppressalis*	JNK	Gut regeneration-related genes	Regulates JAK/STAT pathway to promote midgut regeneration	JNK/JAK/STAT pathway	Inhibiting regeneration enhances Bt toxin susceptibility	[21,48,71]
*S. exigua*	STAT	Carboxylesterases, immune genes	Activates immune responses and detoxification enzyme	JAK/STAT pathway	Enhanced immune evasion and resistance	[21]

**Table 2:** MicroRNA Interactions with Bt Toxin Resistance Mechanisms in Lepidopteran Insects.

miRNA	Species	Population Source	Regulated Object	Regulatory Mechanism	Resistance Phenotype	References
miR-190	*S. frugiperda*	Laboratory strains	β-tubulin	Regulates microtubule stability, affecting midgut cell morphology	Enhances Vip3Aa resistance	[82]
miR-1000	*S. frugiperda*	Laboratory strains	TOR Pathway	TOR activation	Enhances Vip3Aa resistance	[83]
miR-149-3p	*H. armigera*	Field populations	CYP6AE14	CYP450 inhibition mutations cause downregulation	Enhances Vip3Aa resistance	[84]
miR-310	*P. xylostella*	Laboratory strains	Direct ABCC2 mRNA 3′ UTR targeting	Phosphorylation-dependent suppression of receptor genes via MAPK interaction	Enhances Cry1Ac resistance	[85]
miR-2b-3p	*P. xylostella*	Laboratory strains	*PxTrypsin-9*	Trypsin-9 CDS repression	Enhances Cry1Ac resistance	[81]
circRNA_0023	*P. xylostella*	Field populations	miR-203-3p	miRNA sponge	Enhances Cry1Ac resistance	[86]
miR-252	*S. litura*	Field populations	GSTs	GST upregulation	Enhances Cry2Ab resistance	[87]
miR-8510a-3p	*Pectinophora gossypiella*	Field populations	*PxABCG3*	*PxABCG3* CDS upregulation	Enhances Cry1Ac resistance	[7]
miR-7322-5p	*C. suppressalis*	Laboratory strains	p38*/Hsp19*	p38 dephosphorylation *Hsp19* destabilization	Enhances Cry1Ca resistance	[88]
miR-4668	*O. furnacalis*	Field populations	JNK Pathway	JNK phosphorylation inhibition	Enhances Cry1Fa resistance	[89]
miR-3059	*H. virescens*	Not specified	PGRP-LC	Immune receptor suppression	Indirect resistance	[90]
miR-998-3p	*Multiple* spp.	Laboratory strain	ABCC2	ABCC2 3′UTR inhibition	Enhances Cry1Ac resistance	[13]

## Data Availability

No new data were created or analyzed in this study.
